# Central venous versus short midline catheter in difficult intravenous access patients: a randomised clinical pilot trial

**DOI:** 10.1136/bmjopen-2025-113575

**Published:** 2026-02-12

**Authors:** Carl Mellander, Stefanie Seifert, Fredrik Hammarskjöld, Knut Taxbro

**Affiliations:** 1 Department of Biomedical and Clinical Sciences, Linköping University, Linköping, Sweden; 2 Department of Anaesthesia and Intensive Care Medicine, Ryhov County Hospital, Jönköping, Sweden; 3 Futurum Academy of Health and Care, Jönköping, Sweden; 4 Helicopter Emergency Medical Service, Gothenburg, Sweden

**Keywords:** Hospitals, Person-Centered Care, Feasibility Studies, Randomized Controlled Trial, INTENSIVE & CRITICAL CARE, Adult anaesthesia

## Abstract

**Background:**

Patients with difficult intravenous access (DIVA) are at increased risk of delays, discomfort and complications due to multiple failed intravenous access attempts. However, evidence comparing commonly used alternatives, short midline catheters (SMLs) and central venous catheters (CVCs) in this population is limited.

**Objective:**

To evaluate the feasibility of a larger randomised controlled trial comparing SMLs with CVCs in DIVA patients using predefined feasibility outcomes.

**Design:**

This trial was a pragmatic, open-label, single-centre, randomised controlled pilot trial with 1:1 randomisation. Participants were recruited from January to August 2025 with follow-up until September 2025.

**Setting:**

Ryhov County Hospital, Jönköping, a teaching county hospital in Sweden.

**Participants:**

Adult patients (≥18 years) with DIVA, requiring intravenous therapy for 4–29 days.

**Interventions:**

Patients received either a 10-cm SML in the upper arm or a single- or double-lumen CVC in the jugular or subclavian vein.

**Primary and secondary outcome measures:**

Primary outcomes were feasibility criteria: eligibility, recruitment, retention, adherence, missing data and skin puncture attempts. Secondary outcomes included insertion and dwell time, and catheter complications (infection, thrombosis and malfunction).

**Results:**

Of 73 patients screened, 40 (55%) were eligible and 30 (75%) (15 males (50%); median (IQR) age, 73 (61–82) years) were randomised to receive SML (n=15) or CVC (n=15). Three patients in the SML group were not included in the data analysis due to one failed insertion and two incomplete follow-ups. Retention (93%), adherence (97%) and missing data (0%) fulfilled predefined thresholds. The only criterion not met was the number of skin puncture attempts, with 52% of patients requiring two or more. Median catheter dwell time was 5.5 days for SML and 4.0 days for CVC. Complication rates per 1000 catheter days were 101.4 for SML versus 9.1 for CVC, primarily due to a higher rate of malfunction in SML (58% versus 7%). No infections or thromboses were observed.

**Conclusion:**

This pilot trial met all but one feasibility criterion, demonstrating that a larger randomised controlled trial is achievable. The findings highlight practical challenges, particularly related to puncture attempts and catheter performance, that should be addressed in the design of a definitive trial.

**Trial registration number:**

NCT06719869.

**STRENGTHS AND LIMITATIONS OF THIS STUDY**

- The study was designed as a randomised controlled pilot trial to assess the feasibility of a future multicentre trial comparing short midline catheters and central venous catheters in difficult intravenous access patients.
- The study used clear, predefined feasibility criteria (eligibility, recruitment, retention, adherence, missing data and puncture attempts), allowing objective evaluation of trial processes.
- The pilot sample size was limited and not powered to detect differences in clinical outcomes.
- The study was single centre and operator experience was not systematically measured, which may limit generalisability and introduce performance variation between clinicians.

## Introduction

 Intravenous access is essential for most hospitalised patients, with about half requiring a peripheral venous catheter for medication administration or blood sampling.^1^ While establishing peripheral intravenous access is a routine procedure, it can be technically demanding in certain groups of patients, giving rise to the clinical problem known as difficult intravenous access (DIVA). DIVA is typically characterised as cannulation failure of at least two clinicians on two or more attempts using conventional techniques, lack of visible or palpable veins or a documented history of DIVA.^2^ Reported prevalence varies widely (6%–88%) due to differing definitions, with risk factors, including diabetes, intravenous drug use, female sex, chronic illness, obesity and malnutrition.^3 4^ Establishing access in this group can be particularly challenging, often resulting in multiple puncture attempts, procedural and treatment delays and patient discomfort.^5^,^9^

Beyond procedural challenges, repeated failed intravenous attempts can have significant effects on patient well-being. Qualitative research has shown that harmful patient experiences persist and that patients often feel vulnerable when repeated cannulation attempts occur without clear communication, coordination or strategy.^5 6^ Furthermore, unsuccessful insertions and delays in establishing access may not only cause pain and anxiety but also reduce patients’ trust and satisfaction with care.^6^ Reducing failed insertions and procedural complications can improve patient experience while minimising risk of mechanical complications, infection and delays in treatment.

Current research on the management of DIVA patients has largely focused on improving first-attempt success rates of venous cannulation, developing escalation strategies and implementing algorithms to identify patients at risk.^9^,^12^ These efforts aim to reduce procedural delays, multiple punctures and associated patient harm. However, an equally important aspect of a successful intravenous access strategy is the choice of vascular access device. Selecting the most appropriate device for each patient not only influences procedural success but also affects patient safety through complication rates, dwell time and overall experience of care. Adequate device selection is particularly important in patients requiring access for longer than 3 days, where suboptimal choices can lead to repeated insertion attempts.

Alternatives for DIVA patients in need of vascular access for longer than 3 days include, but are not limited to, short midline catheters (SMLs), central venous catheters (CVCs), standard-length midline catheters (MCs) and peripherally inserted central catheters (PICC). SMLs are 6–15 cm peripheral catheters terminating below the axilla,^13 14^ while CVCs are inserted into central veins and terminate in the superior vena cava, right atrium or inferior vena cava. PICCs are long, peripherally inserted catheters that terminate in central veins, while standard-length midlines are longer than 15-cm peripheral catheters terminating in the axillary or subclavian vein; both have been compared head-on in previous meta-analyses and randomised controlled trials comparing their effectiveness and safety.^15^,^18^ Generally, MCs have lower rates of catheter-related infections, such as catheter-related bloodstream infection (CRBSI) compared with PICCs. However, MCs are more frequently removed prematurely or replaced with new MCs or other catheters, likely reflecting a higher risk of mechanical complications.^15^,^19^

Comparisons between SMLs and CVCs are currently based on observational studies. CVCs generally provide reliable central access, but they carry risks, such as arterial puncture, haematoma and pneumothorax.^20 21^ Furthermore, CRBSI may be more common in CVCs compared with SMLs.^22^,^24^ Therefore, SMLs may be a safer option and are recommended by the Michigan Appropriateness Guide for Intravenous Catheters guidelines for patients requiring vascular access for more than 5 days,^13^ yet observational data suggest higher rates of mechanical complications and thrombosis compared with CVCs.^19 22^ Reported dwell times for SMLs vary substantially between studies, whereas CVCs typically remain in place longer.^19 22 25^ The discrepancies in dwell time are likely due to differences in clinical indications and catheter-related outcomes. Similar to MCs, SMLs tend to be removed prematurely.^26 27^ Some patients are reported to require multiple SMLs during one admission, some requiring a CVC after their SML.^19 28^ No randomised controlled trials have directly compared, either standard length or short, MCs to CVC in patients with DIVA. Therefore, the choice of device is currently guided largely by local tradition, observational data and expert opinion.

This pilot trial assessed the feasibility ahead of a larger randomised controlled trial (RCT) comparing SMLs and CVCs in DIVA patients. Feasibility outcomes included eligibility, recruitment, retention and attrition, adherence, missing data and skin puncture attempts.

## Methods

Swedish Ethics Review board approval was obtained on 18 September 2024 prior to trial start (Dnr 2024-05700-01). The trial was registered at the Swedish Medical Products Agency (CIV-24-08-048798) and ClinicalTrials.gov (NCT06719869) prior to its commencement. The trial was conducted in accordance with the principles of the Helsinki Declaration as well as the Consolidated Standards of Reporting Trials extension guidelines for randomised pilot and feasibility trials.^29^ Patients or the public were not involved in the design, conduct or reporting of this trial.

### Trial design

This was a pragmatic, open-label, single-centre, parallel-group, randomised controlled pilot at Ryhov County Hospital, Jönköping, Sweden.

### Participants

Patients aged 18 years or older with one or more DIVA criteria fulfilled, in need of intravenous access for 4–29 days, were eligible for inclusion. DIVA criteria were defined as: ≥2 failed attempts by two clinicians, or no visible or palpable veins, or a documented history of difficult access. Patients were screened for eligibility when ward staff contacted the difficult vascular access service (integrated in the Department of Anaesthesia and Intensive Care Medicine), as per clinical routine. Exclusion criteria were ongoing chemotherapy, administration of hyperosmolar (≥600 mOsm/L) or, otherwise, peripherally incompatible solutions, cognitive impairment and inability to communicate in Scandinavian languages. Written informed consent was required of all patients prior to study inclusion. The numerical feasibility thresholds used in this pilot were pragmatic benchmarks informed by prior trial experience to assess the practical achievability of a subsequent fully powered RCT, rather than guideline-based standards. Given the heterogeneity of pilot trials and the absence of standardised feasibility thresholds, context-specific benchmarks were considered the most appropriate approach for assessing feasibility.

### Interventions

Patients were randomised to receive either the intervention, a 10-cm PowerGlide Pro MC, 20G (Becton Dickinson), or the control, a single (Certofix MonoV) or double-lumen (ArrowguardBlue Plus) CVC inserted by an anaesthesiologist (resident or attending) under ultrasound guidance under maximal sterile precautions.^30^ The insertion site was disinfected with 0.5% chlorhexidine in 70% alcohol and allowed to dry. CVCs were secured with monofilament sutures and the site dressed with a semipermeable dressing (Tegaderm HP; 3M Healthcare). SML insertion was performed under sterile conditions (cap, mask, drape and gloves) by either a nurse anaesthetist or an anaesthesiologist (resident or attending). The insertion site was disinfected with 0.5% chlorhexidine in 70% alcohol and allowed to dry. SML was secured with a StatLock device and semipermeable dressing (Tegaderm HP; 3M Healthcare). For both catheters, choice of insertion site was at the clinician’s discretion. Catheters were controlled three times per day by the ward nurses. Controls included inspection of dressing, puncture site and the catheter itself. Catheter function was assessed and flushed with a minimum of 20 mL saline solution daily and in conjunction with use. Dressings were replaced every third day or earlier if needed. Extension tubing, injection valves and three-way connectors were changed every third day or earlier if needed. For SML, the StatLock Stabilization device was replaced every seventh day or earlier if necessary.

### Outcomes

Primary outcomes were related to feasibility, which was defined as eligibility (>50% of screened patients considered eligible), recruitment (>70% of eligible patients consenting to participate), retention and attrition (<10% of patients lost to follow-up, including withdrawals), adherence (>80% of enrolled patients receiving their randomised intervention), missing data (<10% of enrolled patients with missing data) and skin puncture attempts (<20% of enrolled patients requiring ≥2 skin puncture per insertion). Due to the absence of a standardised definition for skin puncture attempts, the assessment was left to the discretion of each individual inserter.

Secondary outcomes were insertion time, as defined by time from preparing the skin to applying dressing, and dwell time, as defined by time from insertion to catheter removal, measured in minutes and days, respectively. Catheter complications (infection, thrombosis and mechanical failure) were assessed objectively and through clinical expertise in accordance with routine practice. Infection was defined as a positive culture from the catheter tip or presence of clinical signs suggesting catheter-related infection (ie, pus, swelling and pain) or blood cultures indicating catheter-related bacteraemia.^31^ Catheter-related thrombosis was defined as a radiologically confirmed formation of a thrombus in a deep vein anatomically associated with a venous catheter, following clinical suspicion.^32 33^ No routine imaging or laboratory test was performed. Catheter malfunction was defined as catheter failure from accidental removal, dislocation, occlusion, leakage, pain from usage or failure to aspirate blood. Insertion-related complications and reasons for removal were also recorded during follow-up. The data were collected two times per week by a research nurse. If patients were unexpectedly discharged in between follow-ups, retrospective chart reviews were conducted. Patient baseline data and insertion data were collected at enrolment.

### Randomisation

Patients were randomised by the screening nurse anaesthetist using StudyRandomizer (studyrandomizer.com) in a 1:1 ratio with a block size of 6. The allocation sequence was concealed from the person performing the randomisation. Due to the visible nature of the interventions, blinding of the intervention was not possible.

### Statistical analysis and sample size

A convenience sample size of 30 was chosen. Descriptive statistics were presented with numbers and proportions, median and IQRs as appropriate. Frequencies and percentages were reported for categorical variables. A Kaplan–Meier curve was used to visualise cumulative complication-free catheter dwell time. All statistical analyses were performed using SPSS (V.29.0, Armonk, NY, USA).

### Patient and public involvement

Patients and/or the public were not involved in the design, or conduct, or reporting or dissemination plans of this research.

## Results

Patients were recruited from 14 January to 28 August 2025, and the trial was completed on 4 September 2025. The recruitment rate was slightly slower than first anticipated. 30 patients were randomised to either SML (n=15) or CVC (n=15). Patients’ median age was 73 years (IQR 61–82), and 50% were male. All patients required intravenous medication and 70% also required repetitive blood sampling. Two or more failed venipunctures and a history of DIVA were the most common DIVA criteria fulfilled (73% and 93%). Patients’ baseline data are reported in table 1.

**Table 1:** Patient demographics and insertion characteristics

Characteristic	SML	CVC	Total
	n=15	n=15	n=30
Sex, n, (%)			
Male	10 (67)	5 (33)	15 (50)
Female	5 (33)	10 (67)	15 (50)
Age, median (IQR), years	73 (65–86)	73 (54–81)	73 (61–82)
Indication for intravenous access: n, (%)			
Intravenous administration of medications	15 (100)	15 (100)	30 (100)
Administration of fluids or electrolyte solutions	6 (40)	5 (33)	11 (37)
Repetitive blood sampling	11 (73)	10 (67)	21 (70)
Other (such as transfusion)	0 (0)	0 (0)	0 (0)
DIVA criteria fulfilled, n, (%)			
≥2 failed venipunctures by two clinicians	9 (60)	13 (87)	22 (73)
No visible or palpable veins	8 (53)	6 (40)	14 (47)
History of DIVA	14 (93)	14 (93)	28 (93)
Ward, n, %			
Medical	6 (40)	5 (33)	11 (37)
Emergency department	3 (20)	2 (13)	5 (17)
Orthopaedic	3 (20)	4 (27)	7 (23)
Surgical	1 (7)	2 (13)	3 (10)
Infectious disease	1 (7)	2 (13)	3 (10)
Oncology	1 (7)	0 (0)	1 (3)
Insertion characteristics			
Failed insertion, n, (%)	1 (7)	0	1 (3)
Physician insertion, n, %	4 (29)	15 (100)	67
Skin punctures, median, (min–max)	1 (1–5)	2 (1–3)	–
Received>2 skin punctures, n, (% of group)	6 (43)	9 (64)	15 (52)
Number of catheter kits opened*, median, (min–max)	1 (1–5)	1 (1)	–
Vein chosen, n			
Internal jugular vein	0	13	–
Subclavian vein	0	2	–
Cephalic vein	1	0	–
Brachial or basilic vein	12	0	–
Unknown	1	0	–
Side, n (%)			
Left	7 (50)	3 (20)	10 (35)
Right	4 (29)	11 (73)	15 (52)
Unknown	3 (21)	1 (7)	4 (14)
Insertion time, median, (IQR), minutes	14 (10–53)	14 (11–23)	NA
Cumulative insertion time of all insertions, minutes	404	243	NA
Insertion complication, n, (%)			
Haematoma	1 (7)	1 (7)	2 (7)
Arterial puncture	0	1 (7)	1 (3)
Arrhythmia	0	1 (7)	1 (3)
Mechanical	1 (7)	0	1 (3)

* Typically required following multiple cannulation attempts and midline guidewire insertion.

CVC, central venous catheter; DIVA, difficult intravenous access; SML, short midline catheter.

**Figure 1:**
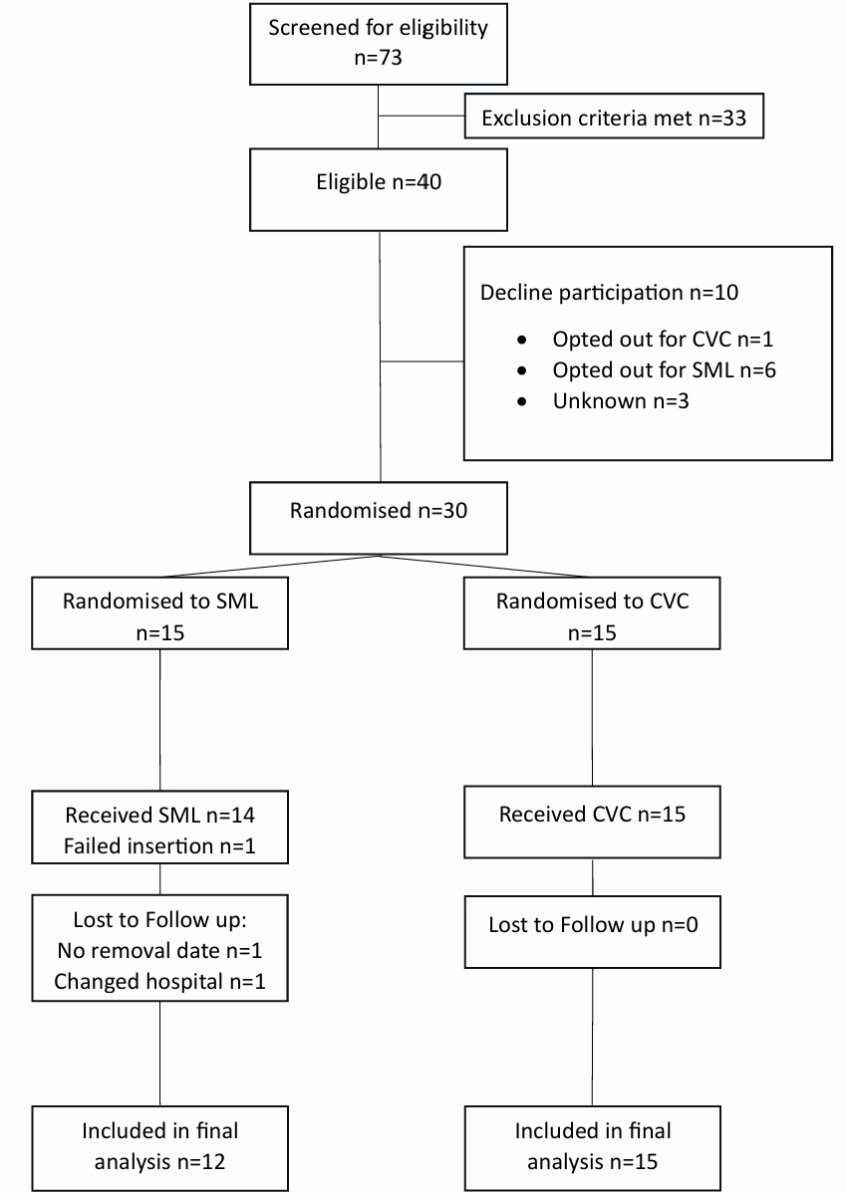
Flowchart of study population. CVC, central venous catheter; SML, short midline catheter.

A total of 73 patients were assessed for eligibility (figure 1). Of these, 33 (45%) were excluded and 40 (55%) were eligible, fulfilling the eligibility benchmark (>50%). Of the 40 eligible patients, 30 (75%) consented to participate (Benchmark >70%). Retention and adherence targets were met, with 2 of 30 patients (7%) lost to follow-up regarding secondary endpoints (<10%) and 29 of 30 (97%) receiving their allocated intervention (>80%). No missing data with regards to the primary outcome were recorded. The only feasibility criteria not achieved were skin puncture attempts, with 15 of 29 patients (52%) requiring ≥2 punctures (benchmark <20%). Fulfilled predefined feasibility criteria are reported in table 2.

**Table 2:** Feasibility criteria

Criteria	Predefined benchmark	Observed benchmark
Eligibility*	>50%	55% (40 of 73)
Recruitment†	>70%	75% (30 of 40)
Retention and attrition‡	<10%	7% (2 of 30)
Adherence§	>80%	97% (29 of 30)
Missing data¶	<10%	0% (0 of 30)
Skin puncture attempts**	<20%	52% (15 of 29)

* Assessed by number of screened patients considered eligible.

† Assessed by number of eligible patients consenting to participate in trial.

‡ Assessed by number of recruited patients lost to follow-up, including withdrawals.

§ Assessed by number of enrolled patients receiving their randomised intervention.

¶ Assessed by number of enrolled patients with any missing data regarding feasibility criteria.

** Assessed by number of enrolled patients requiring ≥2 skin puncture attempts per insertion.

All CVCs were inserted by a resident or attending anaesthesiologist, compared with 29% of SMLs. Median puncture attempts were one (1–5) for SMLs and two (1–3) for CVCs. Insertion time was similar (median 14 min (IQR SML 10–53 and CVC 11–23)), though cumulative insertion time was longer for SMLs (404 vs 243 min). Median dwell time was 5.5 days (IQR 2–9) for SMLs and 4.0 days (IQR 3–10) for CVCs. Procedural characteristics are listed in table 1.

**Figure 2:**
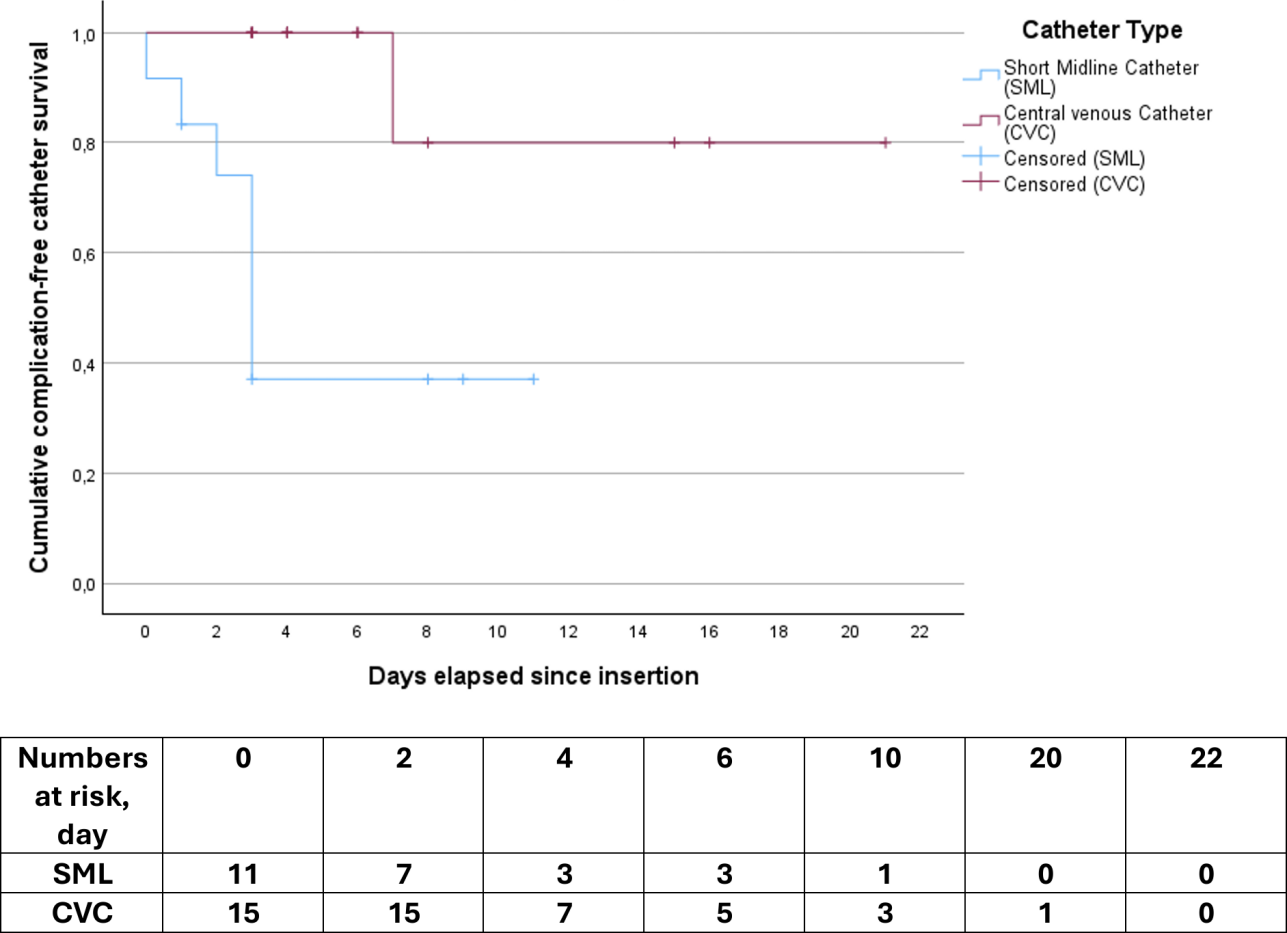
Kaplan–Meier curve displaying cumulative complication-free catheter survival in days.

Complication rates per 1000 catheter days were 101.4 for SMLs and 9.1 for CVCs, respectively. No infections or thromboses were diagnosed. Catheter malfunction was more common in SMLs (58% vs 7%). Catheter dwell times and complications are shown in table 3. Complication-free catheter dwell time is reported in figure 2.

**Table 3:** Dwell time, removal and complications

	SML	CVC	Total
	n=12	n=15	n=27
Catheter dwell time, median, (IQR), days	5.5 (2–9)	4.0 (3–10)	5.0 (3–9)
Cumulative total dwell time, days	69	110	179
Complications per 1000 catheter days	101.4	9.1	NA
Catheter-related infection, n, (% of group)	0	0	0
Catheter-related thrombosis, n, (% of group)	0	0	0
Catheter malfunction, n, (% of group)	7 (58)	1 (7)	8 (30)
Reason for removal, n (% of group)			
No longer needed	8 (67)	14 (93)	22 (81)
Complication	4 (33)	0	4 (15)
Other	0	1 (7)	1 (4)

CVC, central venous catheter; SML, short midline catheter.

## Discussion

To the best of our knowledge, this is the first clinical trial to explore the feasibility of a larger randomised controlled trial comparing SML with CVC in patients with DIVA. All but one of the predefined feasibility criteria were fulfilled, with skin puncture attempts being the only criterion not being met.

Screening was successful, with 55% of eligible patients ultimately included. A substantial proportion of patients were not enrolled due to ward personnel being uncertain about the anticipated duration of vascular access. In such cases, staff often opted for devices perceived as suitable for shorter dwell times, rather than committing to SML or CVC.

Recruitment criteria were fulfilled. Importantly, the most common reason for patients declining participation was a preference for receiving SML rather than a CVC. A possible reason for this might be the perceived invasiveness of receiving a catheter inserted in the neck area rather than the upper arm.

The number of skin punctures was higher than anticipated in the CVC group. We believe that this was mainly due to the absence of a standardised definition of skin puncture attempt, which likely led to over-reporting. The lack of a universally accepted international definition limits comparability between trials and compromises the reliability of this outcome measure. Therefore, despite not fulfilling all predefined feasibility criteria, we assess that a larger randomised controlled trial is feasible. In perspective, prior randomised trials synthesised in a systematic review and meta-analysis indicate that ultrasound guidance increases first-attempt overall cannulation success but does not significantly reduce the number of attempts.^12^ This trial had only one failed insertion, yet 52% of patients were recorded with two or more punctures. Our results align with previous findings and suggest that observed differences may reflect heterogeneity in definitions and documentation rather than actual technical insufficiency. Accordingly, a definitive multicentre RCT should predefine what constitutes an attempt in order to get an accurate measure of procedural difficulty and allow meaningful comparisons between devices.

The cumulative insertion time was greater in the SML group compared with the CVC group (404 vs 243), despite the two groups having a similar median insertion time (14 min, (IQR) SML 10–53 and CVC 11–23). This discrepancy may be explained by variability in personnel experience during the procedures and by differences in individual patient characteristics.^34 35^ In addition, the small sample size of this pilot study is likely to have contributed to the observed variation. In previous studies, median insertion times for SML vary from 5 to 10 min and as for CVC about 12 min in an ICU setting.^16 21 36^

Our trial reported similar dwell time results for both SML and CVC. There were no reported infections or thrombosis either. We observed a high frequency of catheter malfunction in the SML group, largely due to failure to aspirate. Similar results regarding catheter malfunction have been reported previously.^36^ Although failure to aspirate might be considered a minor concern in patients with easily accessible veins, where additional venipunctures can be performed without difficulty, it can have significant implications for individuals with DIVA. Aspiration failure often leads to repeated cannulation attempts and increased patient discomfort. Consequently, it is crucial that the catheter performs reliably for both drug administration and blood collection. The intravenous administration of medications could still be made in some SMLs, despite the failure to aspirate blood that resulted in many patients keeping their SML, a finding also similar to previous studies.^19 22 37^ To improve the trial’s accuracy, a future trial protocol should separate ‘failure to draw blood’ from the ‘catheter malfunction’ variable. This distinction would allow for a more precise and accurate comparison of the catheters. Additionally, recording the need for any extra venipunctures or other vascular access devices during the same hospital stay would be a valuable metric, as it could serve as a reliable measure of the catheter’s overall functionality and patient-centred aspects of care.

The higher rate of mechanical malfunction observed among SMLs may partly reflect differences in design and anatomical positioning rather than patient characteristics or operator performance alone. SMLs terminate in smaller, more superficial veins where movement of the arm, dressing tension and variations in vessel diameter can influence catheter stability and flow dynamics.^38^,^40^ In contrast, CVCs are placed in large central veins with larger luminal diameters and less mechanical stress, which likely contributes to their lower rate of occlusion and dislodgement.

### Generalisability

The methods and findings are generalisable within similar healthcare systems and settings. Nevertheless, a multicentre trial would be necessary for a future RCT to ensure broader external validity of study results.

### Limitations

This pilot trial has some limitations that should be noted. First, experience with CVCs is generally more extensive in our clinic, while SMLs were introduced much later, which may have influenced both staff proficiency and patient outcomes. Second, we did not measure any operator-related variables beyond profession (anaesthesiologist or nurse). Factors, such as operator experience, procedural volume and self-reported confidence, have previously been associated with improved success rates and less complication risks, particularly in more technically challenging insertions.^34 41 42^ The absence of these measures limits our ability to determine whether observed differences between groups were attributable to device performance or operator proficiency. However, with a larger sample size in a multicentre trial, the influence of individual operator differences would likely be less pronounced, providing a more accurate assessment of the devices themselves.

Third, one challenge concerned the biweekly follow-ups. Since patients could be discharged or have their catheter removed between follow-ups, chart reviews were sometimes necessary. This may have introduced inaccuracies regarding when catheters were removed or the reasons for removal, partly due to variations in ward personnel’s experience. Due to the nature of a randomised controlled trial, this likely does not affect internal validity but could affect study reliability, which is a drawback of the pragmatic approach in trial design.

There are several possible adjustments to address these issues. One option would be to increase the number of follow-up visits to reduce the challenges described above. Alternatively, the current design could be maintained, with the assumption that a larger study population in the future main trial would help balance differences arising from individual assessments by ward personnel.

Finally, we used 0.5% chlorhexidine in 70% alcohol for skin antisepsis according to current national recommendations. However, recent guidelines suggest 2% chlorhexidine in 70% alcohol, limiting the generalisability of our results to centres using the more concentrated solution of skin antisepsis.

### Future research

Future studies should aim to address the existing gaps in evidence regarding vascular access in patients with DIVA. Previous clinical trials have primarily focused on improving escalation pathways, prediction tools and techniques aimed at increasing first-attempt cannulation success.^9^,^1243^ However, to the best of our knowledge, relatively few randomised studies have been conducted to determine the most appropriate vascular access device for this patient group.^44^ This is notable since clinical trials in other populations suggest that device choice can influence complication rates, dwell time and, in extension, the need for repeated insertions. Future randomised clinical trials comparing more than SMLs and CVCs in DIVA patients may be required to provide a more comprehensive understanding of how different vascular access devices influence clinical outcomes and patient safety.

## Conclusion

This randomised pilot trial demonstrated the feasibility of a larger trial comparing SML and CVC in patients with DIVA. All but one feasibility criterion was met, indicating that a larger, adequately powered trial is achievable with minor protocol adjustments. Descriptive data suggested more catheter malfunctions in the SML group, while insertion and dwell times were similar. A larger trial is needed to confirm differences in complications and outcomes.

## Data Availability

Data are available on reasonable request.
