# Supplementary material for: Central venous versus short midline catheter in difficult intravenous access patients: a randomised clinical pilot trial
Source: BMJ Open. 2026 Feb 12;16(2):e113575. doi: 10.1136/bmjopen-2025-113575 (PMC12911735; doi:10.1136/bmjopen-2025-113575)
Supplement: Supplementary file 1 [file bmjopen-2025-113575.reviewer_comments.pdf]

PEER REVIEW HISTORY

BMJ Open publishes all reviews undertaken for accepted manuscripts. Reviewers are asked to complete a checklist review form and are provided with free text boxes to elaborate on their assessment. These free text comments are reproduced below.

ARTICLE DETAILS

Title (Provisional)

Central Venous versus short Midline Catheter in Difficult Intravenous Access Patients – A Randomised Clinical Pilot Trial

Authors

Mellander, Carl; Seifert, Stefanie; Hammarskjöld, Fredrik; Taxbro, Knut

---

VERSION 1 - REVIEW

Reviewer 1

Name Gidaro, Antonio

Affiliation Ospedale Luigi Sacco-Polo Universitario, Department of Biomedical and Clinical Sciences

Date 26-Nov-2025

COI None

Due to significant methodological flaws, misclassification, protocol inconsistencies, and deviation from international standards, this manuscript is not suitable for publication in its current form. A complete redesign and transparent reporting are necessary before considering a multicenter RCT.

1) Inconsistent Dates Between the Manuscript and ClinicalTrials.gov

• The manuscript states prospective registration and recruitment from January to August 2025, with completion by September 4, 2025.

• ClinicalTrials.gov (NCT06719869) shows First Posted December 6, 2024, last updated November 18, 2025, with a status of “Completed.” Earlier versions listed estimated completion dates for April–May 2025, which do not match the actual recruitment period. No explanation is provided in the manuscript. Impact: Lack of transparency regarding the trial timeline and protocol deviations.

2) Incorrect Term: “Short Midline Catheter”

According to the NAVIGATE position statement (WoCoVA/GloVANet), catheters measuring 6–15 cm that terminate below the axilla are classified as Long Peripheral Catheters (LPC), not short midline catheters. Midlines are longer than 15 cm and end in the axillary or subclavian

vein. The device studied (10 cm) is therefore an LPC, making the title and comparisons misleading. Impact: Misclassification undermines scientific accuracy and clinical interpretation.

3) Clinical Indications Not Consistent with ERPIUP Consensus

ERPIUP clearly distinguishes peripheral access (LPC/midline) for drugs compatible with peripherally inserted devices and central access for vesicants and hyperosmolar solutions. The manuscript does not specify infusion compatibility criteria but compares LPC with CVC for therapies lasting 4–29 days. Impact: Device performance may be affected more by incorrect use than by inherent differences.

See the papers:

• Quici, M.; Martini, E.; Giustivi, D.; Calloni, M.; Cogliati, C.; Taino, A.; et al. Physicochemical Characteristics of Cardiological Drugs and Practical Recommendations for Intravenous Administration: A Systematic Review. Sci. Pharm. 2025, 93, 13. https://doi.org/10.3390/scipharm93010013

• Manrique-Rodríguez, S., I. Heras-Hidalgo, M. S. Pernia-López, A. Herranz-Alonso, M. C. Del Río Pisabarro, M. B. Suárez-Mier, M.A.Cubero-Pérez ,et al., "Standardization and Chemical Characterization of Intravenous Therapy in Adult Patients: A Step Further in Medication Safety." Drugs in R&D 21, no. 1(2021): 39–64, https://doi.org/10.1007/s40268-020-00329-w.

• Nickel,B., L.Gorski, T. Kleidon, A.Kyes, M.DeVries,S.Keogh, B.Meyer,et al., "Infusion Therapy Standards of Practice, 9th Edition," Journal of Infusion Nursing 47, no. 1S Suppl 1 (2024): S1–S285, https://doi.org/10.1097/NAN.0000000000000532.

• Borgonovo F, Quici M, Gidaro A, Giustivi D, Cattaneo D, Gervasoni C, Calloni M, Martini E, La Cava L, Antinori S, Cogliati C, Gori A, Foschi A. Physicochemical Characteristics of Antimicrobials and Practical Recommendations for Intravenous Administration: A Systematic Review. Antibiotics (Basel). 2023 Aug 19;12(8):1338. doi: 10.3390/antibiotics12081338. PMID: 37627758; PMCID: PMC10451375.

• Pittiruti,M.,T.V.Boxtel,G.Scoppettuolo,P.Carr,E.Konstantinou,G.O.Miluy, M.Lamperti,et al.,"European Recommendations on the Proper Indication and Use of Peripheral Venous Access Devices (the ERPIUP Consensus): A WoCoVA Project." The Journal of Vascular Access 24, no. 1(2023): 165–82,https://doi.org/10.1177/11297298211023274.

4) Substandard Skin Antisepsis versus INS 2024

Both groups used 0.5% chlorhexidine in 70% alcohol, while INS 2024 Standards recommend ≥2% chlorhexidine in alcohol for insertion site antisepsis. Impact: Infection outcomes may not apply to settings following current best practices.

5) Unequal Insertion Bundles

CVC insertion used full maximal sterile precautions (cap, mask, gown, gloves, large drape), while LPC insertion lacked a gown and was often performed by non-anesthesiologists. This introduces procedural bias and may explain higher malfunction rates in LPC.

6) Comparison of Infection Risk Bias Due to Antimicrobial CVCs

The CVC arm included Arrowg+ard Blue Plus, impregnated with chlorhexidine and silver sulfadiazine, proven to reduce CRBSI. This advantage is neither disclosed nor controlled for. Impact: The comparison is methodologically unfair.

7) Convenience Sample Without Power Calculation

The study acknowledges no sample size calculation and uses a convenience sample of 30 patients, limiting interpretability even for feasibility endpoints.

8) Non-standard Definitions and Measurement Bias

• “Catheter malfunction” includes the inability to aspirate blood—a common issue with LPC/midlines—artificially inflating malfunction rates.

• The number of skin punctures was left to the operator's discretion, likely leading to over-reporting for CVC. Impact: Primary feasibility outcomes lack reliability.

9) Misalignment with MAGIC Recommendations

MAGIC recommends LPC/midline access for 6–14 days and compatible peripheral infusions. The trial lasts from 4 to 29 days without stratification, and frequent blood draws were done—an approach not advised for LPC.

**Reviewer**	**2**
**Name**	**Schindler, Ehrenfried**
**Affiliation**	**University of Bonn, Anaesthesiology**
**Date**	**01-Dec-2025**
**COI**	**None**

This is an impoortant and relevant study. Few comments:

1 you wrote that the sample size of 30 is chosen as a convenience sample. I could not more than agree that 30 is a nice nand low number. However you might want to think of an more adequte power analyis. you could problably take your device related infection rates as a basis for a more precise sample size calculation

2. the CVC are sutured and the peripheral ones are taped. A suture is an independent risk factor to promote device related infection. Cant you think about to tape or suture both of them?

3. please describe CVC number of lumen. single, double ot more lumen?

**Reviewer**	**3**
**Name**	**Kvolik, Slavica**
**Affiliation**	**University of Osijek, Anesthesiology, Resuscitation, Critical Care and Pain**
**Date**	**15-Dec-2025**
**COI**	**None**

In the manuscript "Central Venous versus short Midline Catheter in Difficult Intravenous Access Patients – A Randomized Clinical Pilot Trial", the authors analyzed experiences from an RCT comparing using CVC and SML in a pilot study.

The paper is correctly written, but the criteria for the outcomes, the feasibility they wanted to examine, were not mentioned at all. It is necessary to better define outcomes. I.e. according to the current description, "thrombosis in connection to the catheter" is thrombosis of the site representing the junction of the infusion system and the catheter itself; insertion related complications are also not described but are important - they can be the cause of repeated punctures, such as inability to place the catheter after aspiration of blood, collapse of the peripheral vein during puncture, formation of hematoma, etc. Also, state how the decision to open a new package was made. Was a new package used every time after failure of puncture? Were the punctures repeated several times with the same needle? Were decisions made to continue the procedure with the same method on the other side or other puncture site, after initial failure of puncture? Please indicate whether the cumulative insertion time refers to all procedures in the group or all attempts in one patient and from when to when it was defined. Does cumulative insertion time also refer to patient positioning, ultrasound examination before starting skin disinfection and when procedure was considered complete?

Given that no catheter-related thrombosis was observed, please indicate whether and when ALL patients had laboratory tests to confirm (increased) D-dimers and whether each patient had ultrasound and/or CT, given that thrombosis is usualy of varying extent, and those related to the catheter are often asymptomatic. Was ultrasound performed in all patients where the blood could not be aspirated to confirm the cause of catheter malfunction?

Please indicate why the specified number of subjects was used and not another?

Given that a pilot study was conducted, which obviously aimed to make a second larger study feasible, it is not stated nor defined how the numerical threshold for individual outcomes was reached (10% - 70%??). It is also unclear how the size of the pilot study was determined so that it could confirm that a larger study is justified. Although this study is small, statistics is not clear.

VERSION 1 - AUTHOR RESPONSE

Reviewer: 1
Dr. Antonio Gidaro, Ospedale Luigi Sacco-Polo Universitario
Comments to the Author:

Due to significant methodological flaws, misclassification, protocol inconsistencies, and deviation from international standards, this manuscript is not suitable for publication in its current form. A complete redesign and transparent reporting are necessary before considering a multicenter RCT.
1) Inconsistent Dates Between the Manuscript and ClinicalTrials.gov
• The manuscript states prospective registration and recruitment from January to August 2025, with completion by September 4, 2025.

Author response: Thank you for addressing this topic – in the first bullet point dates are consistent between Clinical Trials and the manuscript.

• ClinicalTrials.gov (NCT06719869) shows First Posted December 6, 2024, last updated November 18, 2025, with a status of "Completed." Earlier versions listed estimated completion dates for April–May 2025, which do not match the actual recruitment period. No explanation is provided in the manuscript. Impact: Lack of transparency regarding the trial timeline and protocol deviations.

Author response: We acknowledge the discrepancy between the initially estimated and the actual completion dates. At the time of registration, our projected timeline involved a degree of uncertainty, and participant inclusion progressed more slowly than anticipated. As a result, we were unable to complete recruitment before the summer period. We have added a brief clarification to the manuscript to explain that the inclusion rate was slower than expected.

2) Incorrect Term: "Short Midline Catheter"
According to the NAVIGATE position statement (WoCoVA/GloVANet), catheters measuring 6–15 cm that terminate below the axilla are classified as Long Peripheral Catheters (LPC), not short midline catheters. Midlines are longer than 15 cm and end in the axillary or subclavian vein. The device studied (10 cm) is therefore an LPC, making the title and comparisons misleading. Impact: Misclassification undermines scientific accuracy and clinical interpretation.

Author response: Thank you for this highly insightful comment highlighting a well-known topic of debate. Currently, there is no uniformly accepted definition of midline catheters mirrored by several recent publications and product labelling by manufacturers. The WoCoVA/GloVANet – team should be commended for their efforts toward consistent definitions, and we have referred to their statement paper in our manuscript. This dilemma mandates a thorough description of the catheters used in the trial to inform readers on which catheters were compared.

3) Clinical Indications Not Consistent with ERPIUP Consensus
ERPIUP clearly distinguishes peripheral access (LPC/midline) for drugs compatible with peripherally inserted devices and central access for vesicants and hyperosmolar solutions. The manuscript does not specify infusion compatibility criteria but compares LPC with CVC for therapies lasting 4–29 days. Impact: Device performance may be affected more by incorrect use than by inherent differences.

See the papers:

• Quici, M.; Martini, E.; Giustivi, D.; Calloni, M.; Cogliati, C.; Taino, A.; et al. Physicochemical Characteristics of Cardiological Drugs and Practical Recommendations for Intravenous Administration: A Systematic Review. Sci. Pharm. 2025, 93, 13. https://doi.org/10.3390/scipharm93010013

• Manrique-Rodríguez, S., I. Heras-Hidalgo, M. S. Pernia-López, A. Herranz-Alonso, M. C. Del Río Pisabarro, M. B. Suárez-Mier, M.A.Cubero-Pérez ,et al., "Standardization and Chemical Characterization of Intravenous Therapy in Adult Patients: A Step Further in Medication Safety." Drugs in R&D 21, no. 1(2021): 39–64, https://doi.org/10.1007/s40268-020-00329-w.

• Nickel,B., L.Gorski, T. Kleidon, A.Kyes, M.DeVries,S.Keogh, B.Meyer,et al., "Infusion Therapy Standards of Practice, 9th Edition," Journal of Infusion Nursing 47, no. 1S Suppl 1 (2024): S1–S285, https://doi.org/10.1097/NAN.0000000000000532.

• Borgonovo F, Quici M, Gidaro A, Giustivi D, Cattaneo D, Gervasoni C, Calloni M, Martini E, La Cava L, Antinori S, Cogliati C, Gori A, Foschi A. Physicochemical Characteristics of Antimicrobials and Practical Recommendations for Intravenous Administration: A Systematic Review. Antibiotics (Basel). 2023 Aug 19;12(8):1338. doi: 10.3390/antibiotics12081338. PMID: 37627758; PMCID: PMC10451375.

• Pittiruti,M.,T.V.Boxtel,G.Scoppettuolo,P.Carr,E.Konstantinou,G.O.Miluy, M.Lamperti,et al.,"European Recommendations on the Proper Indication and Use of Peripheral Venous Access Devices (the ERPIUP Consensus): A WoCoVA Project." The Journal of Vascular Access 24, no. 1(2023): 165–82,https://doi.org/10.1177/11297298211023274.

Author response: We appreciate the comment and would like to underline the patient group we studied - DIVA patients are typically depleted of peripheral veins potentially separating this patient group from standard patient populations and corresponding recommendations, making central venous access (either by central or peripheral routes) a viable option for drug delivery and blood sampling, even for peripherally compatible infusates. What we see from practice in Scandinavia is that DIVA patients experience repeated Short PVC cannulation during their hospitalization. Given their typically prolonged length of stay, this leads to repeated punctures, further vascular depletion, and considerable patient discomfort—issues we aim to address by identifying better solutions that align with patient needs and clinical realities.

We have revised the manuscript to clarify that the use of any peripherally incompatible infusate was an exclusion criterion.

4) Substandard Skin Antisepsis versus INS 2024
Both groups used 0.5% chlorhexidine in 70% alcohol, while INS 2024 Standards recommend ≥2% chlorhexidine in alcohol for insertion site antisepsis. Impact: Infection outcomes may not apply to settings following current best practices.
Author response: Thank you for addressing the important aspect of skin antisepsis – the use of 0.5% CHG in 70% alcohol is aligned with current national standards in Sweden, although the INS recently published new guidelines, these have yet to be disseminated into clinical practice. We have added this to the limitations section to better inform readers.

5) Unequal Insertion Bundles
CVC insertion used full maximal sterile precautions (cap, mask, gown, gloves, large drape), while LPC insertion lacked a gown and was often performed by non-anesthesiologists. This introduces procedural bias and may explain higher malfunction rates in LPC.

Author response: We understand and appreciate this comment; however the insertion procedures align with current insertion standards thereby reflect real-world practice supporting the pragmatic study design we aim for.

6) Comparison of Infection Risk Bias Due to Antimicrobial CVCs
The CVC arm included Arrowg+ard Blue Plus, impregnated with chlorhexidine and silver sulfadiazine, proven to reduce CRBSI. This advantage is neither disclosed nor controlled for. Impact: The comparison is methodologically unfair.

Author response: We respectfully disagree with this comment, again all catheters used are standard practice and we would have major ethical concerns by using uncoated catheters for the sole purpose of the trial. Again, reflecting our pragmatic study design.

7) Convenience Sample Without Power Calculation
The study acknowledges no sample size calculation and uses a convenience sample of 30 patients, limiting interpretability even for feasibility endpoints.

Author response: We confirm (as stated in the manuscript) that we have used a convenience sample in the pilot trial. Although formal sample size calculations are generally not a requirement in pilots, we believe that we are transparent in this matter (BMC Med Res Methodol. 2013 Aug 20:13:104.doi: 10.1186/1471-2288-13-104).

8) Non-standard Definitions and Measurement Bias
• "Catheter malfunction" includes the inability to aspirate blood—a common issue with LPC/midlines—artificially inflating malfunction rates.

Author response: Thank you for bringing up this important feature associated with LPC/SML/MLs – in the patient cohort of DIVA patients, we firmly believe that inability to withdraw blood from the catheter is highly relevant. Among hospitalised DIVA-patients,

failure to draw blood will inevitably lead to additional (and likely difficult and painful) venipunctures. Therefore, rather than inflating malfunction rates, we aim to report actual catheter malfunction rates providing a true comparison across the two catheter types.

• The number of skin punctures was left to the operator's discretion, likely leading to over-reporting for CVC. Impact: Primary feasibility outcomes lack reliability.

Author response: We acknowledge this limitation. As with several other insertion-related variables, the number of skin punctures was based on clinician self-report, which introduces the possibility of reporting bias in both groups. This approach may have contributed to over-reporting, particularly for CVC procedures, and we agree that this affects the reliability of the primary feasibility outcomes. We have addressed this issue in the manuscript (page 11)

9) Misalignment with MAGIC Recommendations
MAGIC recommends LPC/midline access for 6–14 days and compatible peripheral infusions. The trial lasts from 4 to 29 days without stratification, and frequent blood draws were done—an approach not advised for LPC.

Author response: We acknowledge the MAGIC recommendations; and the original MAGIC document from 2015 does explicitly take DIVA-patients into consideration. MAGIC prefer PICCs in DIVA patients when the anticipated duration exceeds 14 days but acknowledge that midlines (defined as peripherally inserted catheters between 7,5 to 25 cm in length usually inserted above the antecubital fossa) are approved for longer use if clinically appropriate. In clinical practice, we believe anticipation of catheter duration among DIVA patients poses a challenge. We also thank the reviewer for bringing up the point of stratification of dwell-time, which we will consider during our full-scale RCT.

Reviewer: 2
Prof. Ehrenfried Schindler, University of Bonn
Comments to the Author:

This is an impoortant and relevant study.

Author response: Thank you for your support.

Few comments:
1 you wrote that the sample size of 30 is chosen as a convenience sample. I could not more than agree that 30 is a nice nand low number. However you might want to think of an more adequte power analyis. you could problably take your device related infection rates as a basis for a more precise sample size calculation

Author response: The sample size calculation is of paramount importance for the full-scale RCT. We are currently working on this subject for the statistical analysis plan. We are considering a composite endpoint of all major, patient related complications (pneumothorax,

arterial cannulation, infection, thrombosis and complication-free catheter survival) during insertion and dwell-time.

2. the CVC are sutured and the peripheral ones are taped. A suture is an independent risk factor to promote device related infection. Cant you think about to tape or suture both of them?

Author response: We agree that there is observational data suggesting an association between CVC- anchoring sutures and device infection. In this pragmatic trial, we aim to reflect standard treatment as performed in everyday clinical practice. We also believe that the catheter securement question is highly relevant and our group is currently engaged in the planning of a separate multinational platform trial addressing this topic evaluating both infectious and mechanical complications.

3. please describe CVC number of lumen. single, double ot more lumen?

Author response: The CVC types are reported under "Interventions" in the methods section "*…a Single (Certofix MonoV) or Double-lumen (ArrowguardBlue Plus) CVC inserted…*"

Reviewer: 3
Dr. Slavica Kvolik, University of Osijek
Comments to the Author:

In the manuscript "Central Venous versus short Midline Catheter in Difficult Intravenous Access Patients – A Randomized Clinical Pilot Trial", the authors analyzed experiences from an RCT comparing using CVC and SML in a pilot study.

The paper is correctly written, but the criteria for the outcomes, the feasibility they wanted to examine, were not mentioned at all. It is necessary to better define outcomes.

Author response: The primary and secondary outcomes are defined under "outcomes"; *Feasibility included eligibility (>50% of screened patients considered eligible), recruitment (>70% of eligible patients consenting to participate), retention and attrition (<10% of patients lost to follow-up, including withdrawals), adherence (>80% of enrolled patients receiving their randomised intervention), missing data (<10% of enrolled patients with missing data), and skin puncture attempts (<20% of enrolled patients requiring ≥2 skin puncture per insertion)*. For the secondary outcomes, we specifically refer to scholarly sources for definitions.

I.e. according to the current description, "thrombosis in connection to the catheter" is thrombosis of the site representing the junction of the infusion system and the catheter itself; insertion related complications are also not described but are important - they can be the cause of repeated punctures, such as inability to place the catheter after aspiration of blood, collapse of the peripheral vein during puncture, formation of hematoma, etc.

Author response: We thank the reviewer for this insightful comment on catheter-related DVT definitions. To further clarify our definition, we have revised the text from "thrombosis in connection to the catheter" to "a radiologically confirmed thrombus in a deep vein anatomically associated with a venous catheter".

Also, state how the decision to open a new package was made. Was a new package used every time after failure of puncture? Were the punctures repeated several times with the same needle? Were decisions made to continue the procedure with the same method on the other side or other puncture site, after initial failure of puncture?

Author response: Thank you for identifying this unclarity. We chose to introduce this metric since the PowerGlide Pro has an in-built guidewire. Once this has been advanced towards the "target" vein, it cannot be retracted whenever misplaced and therefore another set must be opened for the procedure to continue. We have clarified this in the Table 1 legend.

Please indicate whether the cumulative insertion time refers to all procedures in the group or all attempts in one patient and from when to when it was defined.

Author response: We are uncertain as how to interpret this comment, we apologize for any misunderstanding – in the table 1 columns the cumulative insertion time per group (SML column 2 and CVC column 3, respectively). The insertion time definition is found under "secondary outcomes".

Does cumulative insertion time also refer to patient positioning, ultrasound examination before starting skin disinfection and when procedure was considered complete?

Author response: The insertion time definition is found under "secondary outcomes".

Given that no catheter-related thrombosis was observed, please indicate whether and when ALL patients had laboratory tests to confirm (increased) D-dimers and whether each patient had ultrasound and/or CT, given that thrombosis is usualy of varying extent, and those related to the catheter are often asymptomatic. Was ultrasound performed in all patients where the blood could not be aspirated to confirm the cause of catheter malfunction?

Author response: Thank you for bringing attention to this – we have clarified in the methods-section that imaging was performed only following clinical suspicion and that neither routine imaging nor laboratory test were performed as a part of the pragmatic trial protocol.

Please indicate why the specified number of subjects was used and not another?

Author response: Similar comments are made by reviewers 1 and 2 - Although formal sample size calculations are generally not a requirement in pilots, we believe that we are transparent in this matter (BMC Med Res Methodol. 2013 Aug 20:13:104.doi: 10.1186/1471-2288-13-104). We also believe that a sample size of 30 is within the generally accepted limits required to address our primary (feasibility) endpoints.

Given that a pilot study was conducted, which obviously aimed to make a second larger study feasible, it is not stated nor defined how the numerical threshold for individual outcomes was reached (10% - 70%??). It is also unclear how the size of the pilot study was determined so that it could confirm that a larger study is justified. Although this study is small, statistics is not clear.

Author response: As described in the "Statistical analysis and Sample Size"-section, we did not perform any statistical between-group comparisons. Due to the pilot-study nature of the trial, we aimed at describing pre-defined criteria of feasibility as primary outcome. For secondary endpoints, the sample size is far too small to draw any meaningful statistically sound conclusions. When performing sample size calculations for the full-scale RCT, we will rely on previously reported data on CVC and midline characteristics.

VERSION 2 - REVIEW

**Reviewer**	**1**
**Name**	**Gidaro, Antonio**
**Affiliation**	**Ospedale Luigi Sacco-Polo Universitario, Department of Biomedical and Clinical Sciences**
**Date**	**08-Jan-2026**
**COI**	

Dear Authors and Editor,

After reviewing the revised submission by Mellander et al., this reviewer respectfully recommends rejection of the manuscript in its current form. Despite the authors' revisions, multiple major concerns remain unresolved and continue to compromise the scientific validity, clinical relevance, and methodological integrity of the study.

1. Persistent Device Misclassification

The trial evaluates a 10-cm, peripherally inserted catheter that terminates below the axilla. This device is consistently classified in international nomenclature as a Long Peripheral Catheter (LPC), not a "short midline." The title, rationale, comparative framework, and interpretation therefore remain misleading. The authors acknowledge debate in terminology but do not correct the classification or the manuscript title. This foundational error affects every level of inference and renders the comparative conclusions unsafe.

2. Inadequate Clinical Rationale and Guideline Misalignment

The study compares CVCs to a peripheral device for therapies reported as compatible with peripheral venous access, over a dwell-time window of 4–29 days without stratification. This approach is incongruent with established guidance (e.g., ERPIUP and MAGIC), which clearly reserves central lines for vesicants, hyperosmolar solutions, or indications not suitable for peripheral devices. The authors' justification that DIVA patients have "depleted peripheral veins" is not sufficient to

warrant central catheter use where therapy remains peripheral-compatible. Consequently, the trial's core clinical question is not appropriate, and its external validity is limited.

3. Non-Adherence to International Antisepsis Standards

Both arms employed 0.5% chlorhexidine in 70% alcohol for skin antisepsis. International standards (e.g., INS and SHEA) have recommended ≥2% chlorhexidine in alcohol for catheter insertion site preparation for over a decade. Relocating this discrepancy to the limitations section does not address the fact that infection-related outcomes cannot be generalised to settings compliant with current standards. The choice of a lower-strength antiseptic is a preventable methodological flaw that should have been corrected.

4. Systematic Procedural Bias and Unequal Arms

The CVC arm benefited from maximal sterile precautions and antimicrobial-impregnated catheters (e.g., Arrowg+ard Blue Plus), whereas the peripheral arm did not. These differences introduce arm-specific advantages that bias infection and malfunction outcomes. The authors defend this as "pragmatic," but such asymmetry should be balanced or analytically controlled to permit fair comparison. As presented, the study design favours CVCs and invalidates arm-to-arm equivalence.

5. Insufficient Statistical Planning for Feasibility

The study utilises a convenience sample (n=30) without a priori justification of feasibility thresholds (e.g., recruitment ≥70%, puncture attempts <20%). Even for pilot work, a transparent rationale for benchmarks and a minimal statistical framework are expected. In the absence of these elements, the claim that the study "demonstrates feasibility" lacks credible support.

6. Non-Standard Endpoints and Measurement Bias

The primary complication endpoint "catheter malfunction" aggregates the inability to aspirate blood with mechanical failures. Failure to aspirate is common in LPCs and should be analysed separately as a patient-centred outcome (e.g., additional venipunctures, alternative access) to avoid inflating malfunction rates in one arm. See the recent papers:

- Binda F, Galazzi A, Marelli F, Salinaro G, de Vecchi M, Spagna S, Marcotullio A, Calegari J, Laquintana D, Gambazza S. Long versus conventional peripheral intravenous catheters to reduce venipunctures during hospital stay: A randomized controlled trial. J Vasc Access. 2025 Nov 3:11297298251385595. doi: 10.1177/11297298251385595. Epub ahead of print. PMID: 41185485.
- Bahl A, Drogowski M, Gutta A, Lehman C, Younes E, DiLoreto E, Shen C. Upper Arm Versus Forearm Placement of Long Peripheral Catheters for Blood Sampling: A Randomized Controlled Trial. J Nurs Care Qual. 2025 Oct-Dec 01;40(4):310-317. doi: 10.1097/NCQ.0000000000000857. Epub 2025 Mar 12. PMID: 40073101.

Moreover, skin puncture counts were left to the operator's discretion, likely resulting in overreporting in the CVC group and undermining the reliability of the feasibility endpoints. These definitional choices are non-standard and arm-sensitive, limiting comparability with prior literature.

7. Dwell-Time Window and Escalation Strategy

The dwell-time span 4–29 days is not aligned with appropriateness criteria for LPC/midline access (typically 6–14 days for compatible infusions), and the protocol does not stratify by anticipated duration or infusion characteristics. Including frequent blood draws—discouraged for LPCs—further

reduces the clinical coherence of the comparison. These design features should have been predefined and stratified in accordance with best practice.

Overall Judgement

The revised manuscript does not satisfactorily address the major concerns raised by Reviewer 1 and others. The device misclassification, inappropriate clinical rationale for central access, deviation from internationally accepted antisepsis standards, unequal insertion bundles, unsubstantiated feasibility thresholds, and non-standard endpoint definitions collectively preclude publication at this time. The study's conclusions regarding feasibility and comparative performance are unreliable due to structural design flaws rather than minor, correctable issues.

Recommendation

Reject. If the authors wish to resubmit, a comprehensive redesign will be necessary, including:

- Correct device classification and revision of the title and all related text (LPC vs. "short midline").
- Alignment with guidelines for device selection (ERPIUP/MAGIC): study only patients with indications appropriate to each device; predefine stratification by therapy compatibility and anticipated dwell time.
- Antisepsis compliance with international standards (≥2% chlorhexidine in alcohol) across arms.
- Balanced procedural bundles and removal or analytical control of antimicrobial-impregnated catheter advantages in the CVC arm.
- Predefined, arm-neutral endpoints, separating "failure to aspirate" from true mechanical failure; standardised definitions for "puncture attempts."
- Justified feasibility benchmarks and a minimal sample size rationale appropriate for pilot work.

This recommendation is made to uphold methodological rigor and ensure that any future definitive trials in DIVA patients are ethically justified, clinically appropriate, and aligned with international standards.

**Reviewer**	**2**
**Name**	**Schindler, Ehrenfried**
**Affiliation**	**University of Bonn, Anaesthesiology**
**Date**	**08-Jan-2026**
**COI**	

---

thank you, no further questions

**Reviewer**	**3**
**Name**	**Kvolik, Slavica**

Affiliation **University of Osijek, Anesthesiology, Resuscitation, Critical Care and Pain**

Date **22-Jan-2026**

COI

Dear authors

The answers to my first question were not specific. When asked how you defined the numerical criteria for outcomes - a question was WHY is the criterion >50% of screened patients considered eligible, and for recruitment >70% AND NOT LESS OR MORE? Are the reported values YOUR position or are recommended, accepted values for feasibility or pilot studies (eg some guidelines?)? If you were guided by the criteria used by other authors, it is recommended to refer to another paper, if not, please write why you chose these specific values.

You gave satisfactory answers to my other questions. After reading your article, along with the responses to other reviewers, I believe that after the corrections were made, the paper represents a real-life feasibility study and is suitable for publication.

VERSION 2 - AUTHOR RESPONSE

Reviewer: 2
Prof. Ehrenfried Schindler, University of Bonn
Comments to the Author:

thank you, no further questions

Author response: We thank Reviewer 2 for their assessment and appreciate their contribution.

Reviewer: 1
Dr. Antonio Gidaro, Ospedale Luigi Sacco-Polo Universitario
Comments to the Author:

Dear Authors and Editor,
After reviewing the revised submission by Mellander et al., this reviewer respectfully recommends rejection of the manuscript in its current form. Despite the authors' revisions, multiple major concerns remain unresolved and continue to compromise the scientific validity, clinical relevance, and methodological integrity of the study.

1. Persistent Device Misclassification
The trial evaluates a 10-cm, peripherally inserted catheter that terminates below the axilla. This device is consistently classified in international nomenclature as a Long Peripheral Catheter

(LPC), not a "short midline." The title, rationale, comparative framework, and interpretation therefore remain misleading. The authors acknowledge debate in terminology but do not correct the classification or the manuscript title. This foundational error affects every level of inference and renders the comparative conclusions unsafe.

Author response: We thank the reviewer for highlighting the importance of accurate device classification. We respectfully maintain the use of the term "short midline catheter", as current international nomenclature for 6–15 cm peripherally inserted catheters remain inconsistent across guidelines and published studies. The WoCoVA/ERPIUP consensus explicitly acknowledges that these devices are referred to by multiple names, "long peripheral catheter (LPC), mini-midline, and short midline", noting that this variability has created "misunderstanding into the classification and terminology of VADs" in both research and clinical practice. (J Vasc Access. 2023 Jan;24(1):165-182. doi: 10.1177/11297298211023274). ERPIUP attributes this heterogeneity partly to differences between European and North American terminology and recommends that authors ensure clarity by describing device length, insertion approach and tip location, particularly when terminology varies.

Following these recommendations, our manuscript provides a precise description of the studied device (10 cm, upper-arm insertion, tip below the axilla), allowing readers to accurately situate it within the ERPIUP LPC category while still recognising the widely used term "short midline." Given this acknowledged heterogeneity, we believe retaining the established clinical term while ensuring transparent technical description is the most informative approach for readers.

2. Inadequate Clinical Rationale and Guideline Misalignment
The study compares CVCs to a peripheral device for therapies reported as compatible with peripheral venous access, over a dwell-time window of 4–29 days without stratification. This approach is incongruent with established guidance (e.g., ERPIUP and MAGIC), which clearly reserves central lines for vesicants, hyperosmolar solutions, or indications not suitable for peripheral devices. The authors' justification that DIVA patients have "depleted peripheral veins" is not sufficient to warrant central catheter use where therapy remains peripheral-compatible. Consequently, the trial's core clinical question is not appropriate, and its external validity is limited.

Author response: We thank the reviewer for this important comment. We agree that ERPIUP and MAGIC emphasise the avoidance of central lines when infusates are peripherally compatible, however, both guidelines also acknowledge that device selection must account for patient-specific factors, including anticipated duration, vascular access limitations and the feasibility of blood sampling.

The ERPIUP consensus recommends long peripheral catheters or midlines for DIVA patients, but it also highlights that these devices are heterogeneous and may present practical limitations, including difficulties with reliable blood sampling, which is particularly relevant in DIVA populations who often cannot undergo repeated venipuncture safely or comfortably. This challenge was also noted by Reviewer 1 and represents a key clinical problem our study seeks to address.

Regarding MAGIC, while CVCs are generally reserved for vesicants or incompatible solutions, MAGIC also states that in DIVA patients with short-term access needs (<14 days), CVCs may be preferred over midlines when reliable access is essential, particularly in critically ill patients or when hemodynamic monitoring is needed (Ann Intern Med. 2015 Sep 15;163(6 Suppl):S1-40. doi: 10.7326/M15-0744.). These recommendations are based largely on observational evidence, underscoring the need for randomised trials to determine optimal device selection in DIVA patients, a gap our pilot trial and subsequent fully powered trial hope to address.

3. Non-Adherence to International Antisepsis Standards
Both arms employed 0.5% chlorhexidine in 70% alcohol for skin antisepsis. International standards (e.g., INS and SHEA) have recommended ≥2% chlorhexidine in alcohol for catheter insertion site preparation for over a decade. Relocating this discrepancy to the limitations section does not address the fact that infection-related outcomes cannot be generalised to settings compliant with current standards. The choice of a lower-strength antiseptic is a preventable methodological flaw that should have been corrected.

Author response: We thank the reviewer for this comment. We acknowledge that INS and SHEA recommend ≥2% chlorhexidine in alcohol, however, 0.5% chlorhexidine in 70% alcohol remains the national standard in Sweden, where this pragmatic pilot was conducted. Evidence directly comparing 0.5% versus 2% chlorhexidine is limited and a large RCT demonstrated no significant difference in catheter-related infection rates between concentrations (Infect Control Hosp Epidemiol. 2008 Sep;29(9):847-53. doi: 10.1086/590259.) As infection outcomes were not primary endpoints and no infections occurred, we have appropriately addressed this limitation but cannot retroactively modify trial procedures.

4. Systematic Procedural Bias and Unequal Arms
The CVC arm benefited from maximal sterile precautions and antimicrobial-impregnated catheters (e.g., Arrowg+ard Blue Plus), whereas the peripheral arm did not. These differences introduce arm-specific advantages that bias infection and malfunction outcomes. The authors defend this as "pragmatic," but such asymmetry should be balanced or analytically controlled to permit fair comparison. As presented, the study design favours CVCs and invalidates arm-to-arm equivalence.

Author response: We acknowledge the reviewer's concern regarding procedural differences between groups. As a pragmatic pilot trial, our intent was to reflect real-world practice, where CVCs are routinely inserted using maximal sterile precautions and, at our institution, often include antimicrobial-impregnated catheters, while peripheral devices are inserted under standard sterile conditions. However, this practice reflects everyday clinical life. There are advantages and disadvantages with all catheter types; the key question is which option ultimately provides the safest overall profile. We agree that such differences could influence infection-related or mechanical outcomes, therefore, we made no statistical comparisons between catheter types and explicitly avoided p-values. Our focus was feasibility rather than comparative efficacy.

5. Insufficient Statistical Planning for Feasibility
The study utilises a convenience sample (n=30) without a priori justification of feasibility thresholds (e.g., recruitment ≥70%, puncture attempts <20%). Even for pilot work, a transparent rationale for benchmarks and a minimal statistical framework are expected. In the absence of these elements, the claim that the study "demonstrates feasibility" lacks credible support.

Author response: We thank the reviewer for this comment, which was also raised by Reviewer 1, 2 and 3 in the previous peer-review. As stated in the manuscript, this pilot trial used a convenience sample. Consistent with established methodological guidance, formal sample size calculations are not required for pilot or feasibility studies. (BMC Med Res Methodol. 2013 Aug 20:13:104.doi: 10.1186/1471-2288-13-104)

The numerical feasibility thresholds we used (>50% eligibility, >70% recruitment, <10% attrition, etc.) were pragmatically determined through internal discussion within our research group, informed by experience from previous trials conducted in our clinical setting. These numerical thresholds were chosen as practical feasibility markers based on our local experience, rather than as values set by any guideline and were intended to indicate whether a full RCT would be realistically achievable. Due to the heterogeneity between pilot trials and their varying aims, settings and constraints, benchmarks used by previous pilot studies were not applicable to our local context. For example, some pilot studies (Int J Nurs Pract. 2023 Apr;29(2):e13110. doi: 10.1111/ijn.13110. Epub 2022 Oct 27.) in similar clinical areas report eligibility rates above 90%; however, achieving such a threshold in our setting would imply that more than 90% of all DIVA patients screened at our hospital would require 4–28 days of intravenous access needs, have no cognitive impairment and meet both language and age criteria, an expectation that is not realistic in our clinical practice. We have now stated this explicitly in the manuscript (p. 8).

While benchmark thresholds vary across feasibility research, our study met all but one criterion, supporting the conclusion that progression to a definitive RCT is achievable.

6. Non-Standard Endpoints and Measurement Bias
The primary complication endpoint "catheter malfunction" aggregates the inability to aspirate blood with mechanical failures. Failure to aspirate is common in LPCs and should be analysed separately as a patient-centred outcome (e.g., additional venipunctures, alternative access) to avoid inflating malfunction rates in one arm. See the recent papers:
• Binda F, Galazzi A, Marelli F, Salinaro G, de Vecchi M, Spagna S, Marcotullio A, Calegari J, Laquintana D, Gambazza S. Long versus conventional peripheral intravenous catheters to reduce venipunctures during hospital stay: A randomized controlled trial. J Vasc Access. 2025 Nov 3:11297298251385595. doi: 10.1177/11297298251385595. Epub ahead of print. PMID: 41185485.
• Bahl A, Drogowski M, Gutta A, Lehman C, Younes E, DiLoreto E, Shen C. Upper Arm Versus Forearm Placement of Long Peripheral Catheters for Blood Sampling: A Randomized Controlled Trial. J Nurs Care Qual. 2025 Oct-Dec 01;40(4):310-317. doi: 10.1097/NCQ.0000000000000857. Epub 2025 Mar 12. PMID: 40073101.

Author response: We agree with the reviewer that combining failure to aspirate with mechanical complications is a limitation, particularly as inability to aspirate is known to be more frequent in LPC-type devices/short midline catheters and represents an important patient-centred

outcome. This issue is explicitly acknowledged in our manuscript and will be addressed in the protocol for the fully powered RCT, where “catheter malfunction” will be separated into distinct, predefined components (e.g., aspiration failure, occlusion, dislodgement).

However, we emphasise that the primary aim of this pilot was feasibility, not comparative complication rates and the study was not powered for between-group comparisons. Accordingly, no statistical comparisons were performed and complication data were presented descriptively to inform refinement of outcome definitions for the definitive multicentre trial.

Moreover, skin puncture counts were left to the operator's discretion, likely resulting in overreporting in the CVC group and undermining the reliability of the feasibility endpoints. These definitional choices are non-standard and arm-sensitive, limiting comparability with prior literature.

Author response: We agree that puncture attempts were likely overreported, particularly in the CVC group. However, there is currently no internationally standardised definition of a “skin puncture attempt”, which inherently limits comparability across studies. We have acknowledged this in the discussion as well as its implications. We have also stated this more clearly in the marked manuscript (p. 12).

7. Dwell-Time Window and Escalation Strategy
The dwell-time span 4–29 days is not aligned with appropriateness criteria for LPC/midline access (typically 6–14 days for compatible infusions), and the protocol does not stratify by anticipated duration or infusion characteristics. Including frequent blood draws—discouraged for LPCs—further reduces the clinical coherence of the comparison. These design features should have been predefined and stratified in accordance with best practice.

Author response: We thank the reviewer for this comment. As outlined previously, ERPIUP and MAGIC provide general dwell-time recommendations for LPC-type devices/short midline catheters, but these guidelines are based largely on observational data or expert opinion and do not fully address the complex reality of DIVA patients. In this population, daily blood sampling is a common and necessary part of inpatient care, yet repeated venipuncture is often not feasible. At the same time, the recommended LPC-type devices/short midline catheters may not consistently support reliable aspiration, creating a clinical dilemma. This challenge, as highlighted by Reviewer 1, exposes a current scientific grey zone in vascular access decision-making for DIVA patients.

Our pilot trial was not designed nor adequately powered to compare dwell times or clinical performance between groups, but to assess feasibility of conducting a larger RCT in this population.

Overall Judgement
The revised manuscript does not satisfactorily address the major concerns raised by Reviewer 1 and others. The device misclassification, inappropriate clinical rationale for central access, deviation from internationally accepted antisepsis standards, unequal insertion bundles, unsubstantiated feasibility thresholds, and non-standard endpoint definitions collectively

preclude publication at this time. The study's conclusions regarding feasibility and comparative performance are unreliable due to structural design flaws rather than minor, correctable issues.

Recommendation
Reject. If the authors wish to resubmit, a comprehensive redesign will be necessary, including:

• Correct device classification and revision of the title and all related text (LPC vs. "short midline").
• Alignment with guidelines for device selection (ERPIUP/MAGIC): study only patients with indications appropriate to each device; predefine stratification by therapy compatibility and anticipated dwell time.
• Antisepsis compliance with international standards (≥2% chlorhexidine in alcohol) across arms.
• Balanced procedural bundles and removal or analytical control of antimicrobial-impregnated catheter advantages in the CVC arm.
• Predefined, arm-neutral endpoints, separating "failure to aspirate" from true mechanical failure; standardised definitions for "puncture attempts."
• Justified feasibility benchmarks and a minimal sample size rationale appropriate for pilot work.
This recommendation is made to uphold methodological rigor and ensure that any future definitive trials in DIVA patients are ethically justified, clinically appropriate, and aligned with international standards.

Author response: We appreciate the reviewer's thorough assessment. The recommendations listed have been addressed individually in our detailed responses above. Several points raised involve methodological elements inherent to the pragmatic pilot design or aspects that cannot be retroactively altered (e.g., antisepsis concentration, procedural bundles, sample size). As such, we are unable to repeat or redesign the completed trial. However, we have clarified these limitations transparently and incorporated the reviewer's suggestions into the planning of our fully powered future RCT, where they can be appropriately implemented.

Reviewer: 3
Dr. Slavica Kvolik, University of Osijek
Comments to the Author:

Dear authors
The answers to my first question were not specific. When asked how you defined the numerical criteria for outcomes - a question was WHY is the criterion >50% of screened patients considered eligible, and for recruitment >70% AND NOT LESS OR MORE? Are the reported values YOUR position or are recommended, accepted values for feasibility or pilot studies (eg some guidelines?)? If you were guided by the criteria used by other authors, it is recommended to refer to another paper, if not, please write why you chose these specific values.

Author response: We thank the reviewer for this clarification request which also was requested by Reviewer 1. The numerical feasibility thresholds we used (>50% eligibility, >70% recruitment, <10% attrition, etc.) were pragmatically determined through internal discussion within our research group, informed by experience from previous trials conducted in our clinical setting. These numerical thresholds were chosen as practical feasibility markers based on our local experience, rather than as values set by any guideline and were intended to indicate whether a full RCT would be realistically achievable. Due to the heterogeneity between pilot trials and their varying aims, settings and constraints, benchmarks used by previous pilot studies were not applicable to our local context. For example, some pilot studies (Int J Nurs Pract. 2023 Apr;29(2):e13110. doi: 10.1111/ijn.13110. Epub 2022 Oct 27.) in similar clinical areas report eligibility rates above 90%; however, achieving such a threshold in our setting would imply that more than 90% of all DIVA patients screened at our hospital would require 4–28 days of intravenous access needs, have no cognitive impairment, and meet both language and age criteria, a target that is not feasible in our setting. We have now stated this explicitly in the manuscript. (p. 8)

You gave satisfactory answers to my other questions. After reading your article, along with the responses to other reviewers, I believe that after the corrections were made, the paper represents a real-life feasibility study and is suitable for publication.

Author response: Thank you for your insight and thorough review.
